# Estrogen Receptor-Low Breast Cancer With Sternal Metastasis Presenting as “Stiff Neck” in a Young Female

**DOI:** 10.7759/cureus.99072

**Published:** 2025-12-12

**Authors:** Hinata Nishimura, Yuichiro Mine, Yuichi Takahashi, Gautam A Deshpande, Junichiro Watanabe, Goro Kutomi, Toshio Naito

**Affiliations:** 1 Clinical Training Center, Juntendo University Hospital, Tokyo, JPN; 2 Department of General Medicine, Juntendo University Faculty of Medicine, Tokyo, JPN; 3 Department of Breast Oncology, Juntendo University Juntendo Hospital, Juntendo University School of Medicine, Tokyo, JPN; 4 Department of Breast Oncology, Juntendo University Faculty of Medicine, Tokyo, JPN; 5 Department of General Medicine, Juntendo University, Tokyo, JPN

**Keywords:** diagnostic delay, neck stiffness, physician-patient relations, sternal metastasis, triple-negative breast cancer

## Abstract

A previously healthy 20-year-old Japanese woman was referred to the outpatient clinic for evaluation of right neck stiffness, which had persisted for two months, along with weight loss, malaise, and elevated inflammatory markers. On physical examination, spontaneous pain was noted in the right upper trapezius, along with incident pain around both clavicles and shoulders. Swelling was found around the sternal manubrium, and two palpable masses were present in the right breast. A computed tomography scan of the thorax revealed a low-density, lobulated area in the right breast, a soft-tissue mass in the sternal manubrium, multiple lymphadenopathies, and small nodules in the lungs and liver. Core needle biopsy of the breast mass confirmed estrogen receptor (ER)-low invasive ductal carcinoma, and testing for breast cancer susceptibility gene mutations was negative. Systemic chemotherapy was initiated for the treatment of metastatic triple-negative breast cancer.

Breast cancer causing neck or shoulder pain is rare. The right neck stiffness likely resulted from brachial plexus compression due to bulky right axillary lymphadenopathy, leading to thoracic outlet syndrome. Lung metastasis could also cause referred pain through the vagus nerve. This case presented an atypical manifestation of "stiff neck" associated with thoracic tumors. Notably, the patient initially hesitated to share key symptoms, which may have contributed to a delayed diagnosis. We believe that the rapid progression of her lesions may have heightened her anxiety, which in turn further impaired her communication with the medical staff.

## Introduction

Breast carcinoma with low estrogen receptor (ER) expression has recently been recognized as a distinct biological subtype from other ER-positive breast tumors. The current American Society of Clinical Oncology/College of American Pathologists guidelines define tumors with 1-10% ER expression as ER-low-positive, representing approximately 2-7% of all breast cancers [1,2]. This subtype has attracted attention because of its clinical and pathological similarities to triple-negative breast cancer (TNBC), including its prevalence among patients under 50, higher histological grade, and poorer prognosis compared with ER-high tumors [2].

Breast cancer typically appears painless in early stages, yet presents with pain in advanced stages by involving surrounding structures or metastasizing to distant sites [3]. The most well-known source of symptomatic pain in advanced breast cancer is bone metastasis, which will mainly cause somatic pain [4]. At the same time, invasion along the nervous system can directly damage nerves, causing neuropathic pain [3]. Metastases in the lungs or liver may also produce visceral pain due to tissue destruction and inflammation. As a result, breast malignancy may occasionally present with non-specific pain, as seen in this case, making it easy to overlook without a thorough physical examination and appropriate imaging.

## Case presentation

**Figure 1:**
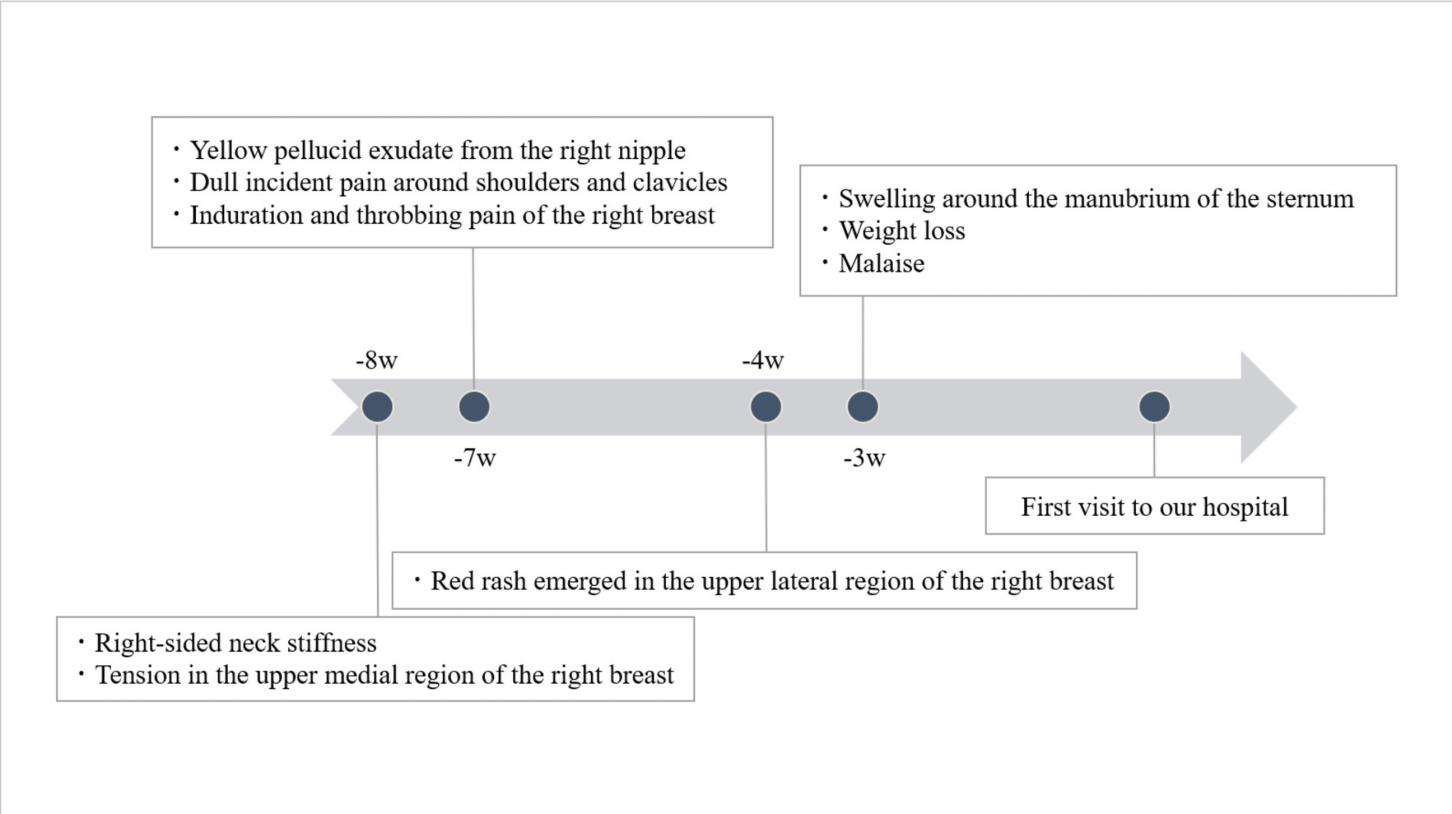
Symptom chronology until the first visit. Retrospectively reported symptoms after the breast masses were identified.

A 20-year-old previously healthy Japanese woman was referred for further examination of persistent right-sided neck stiffness for two months, accompanied by a 3-week history of weight loss, malaise, and elevated white blood cells (9.4×10⁹ /L; normal range: 3.6-8.9 ×10⁹/L) and C-reactive protein of 1.91 mg/dL (normal range: below 1 mg/dL). She had no additional complaints aside from a stiff neck, which gradually evolved into a dull pain radiating to the shoulders and clavicles. Chronology, including other symptoms, is shown in Figure 1.

**Figure 2:**
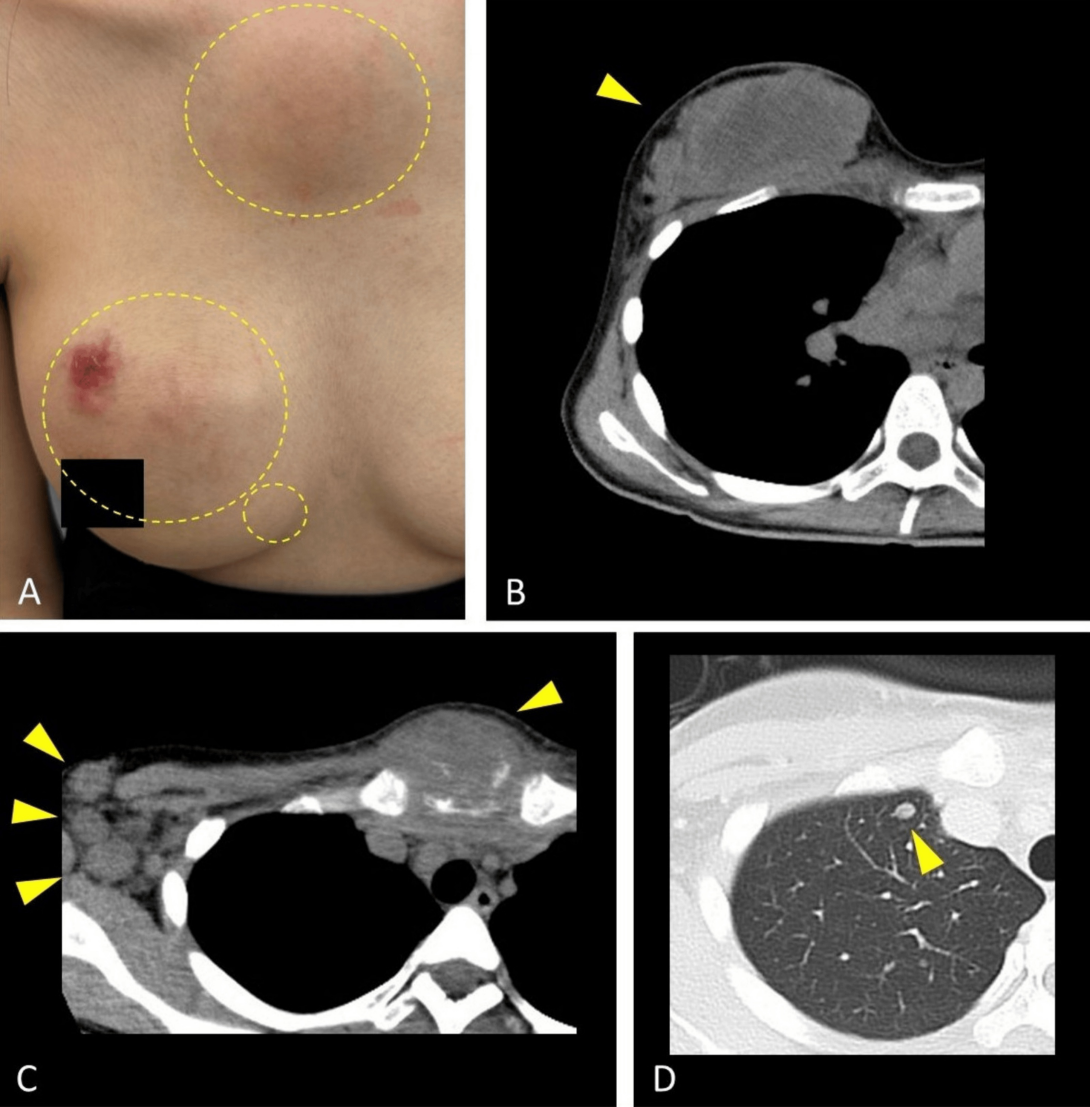
Clinical presentation and imaging findings of metastatic triple-negative breast cancer. (A) Clinical photograph of the right breast showing a primary breast mass (70 mm × 110 mm) with overlying skin erosion, a satellite nodule (20 mm × 15 mm) near the primary lesion, and significant swelling around the sternal manubrium (70 mm × 70 mm) (areas of interest indicated by circles). (B) Axial view of the contrast-enhanced computed tomography scan of the chest demonstrating a lobulated low-density mass in the right breast (arrow heads), indicative of the primary tumor. (C) Axial view of the computed tomography scan demonstrating a soft tissue mass involving the sternal manubrium (arrowhead) with surrounding bony destruction (small arrowheads), along with multiple enlarged right axillary and bilateral supraclavicular lymph nodes (arrowheads).  (D) Axial view of the pulmonary computed tomography scan showing a small nodule in the right lung apex (arrow head), consistent with lung metastasis.

Her Eastern Cooperative Oncology Group performance status was 0, and her family history was unremarkable for malignancy. Physical examination revealed spontaneous pain around the right upper trapezius, and incident pain around the bilateral clavicles and shoulders, but no sensory deficit or numbness. Notably, a swelling over the sternal manubrium, two palpable right breast masses with overlying skin erosion, stony-hard right axillary lymphadenopathy, and serous nipple discharge were observed (Figure 2A). A plain computed tomography scan of the thorax, performed due to a suspicion of breast malignancy, revealed a lobulated low-density lesion in the right breast (Figure 2B), a soft-tissue mass involving the sternal manubrium with adjacent bone destruction, multiple lymphadenopathies (Figure 2C), and small nodules in the lungs (Figure 2D) and liver.

A core needle biopsy of the breast lesion confirmed invasive ductal carcinoma with 5% of estrogen receptor (ER) expression, negative progesterone receptor and human epidermal growth factor receptor 2 expression, and 80% of Ki-67 expression. The BRCA1/2 genetic test revealed no pathogenic variants. Pathology demonstrated PD-L1 positivity using the 22C3 immunohistochemistry assay, with a combined positive score (CPS) of 30. Therefore, based on KEYNOTE-355 criteria, systemic chemotherapy with pembrolizumab, carboplatin, and gemcitabine was initiated 19 days from the first visit as PD-L1-positive metastatic TNBC [5]. Following the initiation of chemotherapy, her neck stiffness improved as the breast lesions decreased in size. The patient initially reported mild nausea and constipation, with no other significant adverse effects.

## Discussion

Among metastatic breast cancer subtypes, TNBC demonstrates the most aggressive clinical course, with a median overall survival of 14.5 months and a 5-year survival rate of 11.3% [6]. Breast cancer in women under the age of 30 demonstrates a high Ki-67 proliferation index, high BRCA1/2 pathogenic variant rates, and low estrogen receptor (ER) expression, contributing to advanced-stage presentation [7]. In this case, the pathology demonstrated 80% Ki-67 expression and 5% ER expression, despite negative BRCA1/2 results. In principle, TNBC is defined by ER expression below 1%; therefore, the pathology of this patient, with an ER expression of 1-10%, is classified as ER-low breast carcinoma. ER-low tumors have been reported to exhibit immune characteristics similar to those of TNBC, including PD-L1 expression, and it has been suggested that managing ER-low tumors in a manner analogous to TNBC, such as incorporating immune checkpoint blockade, may improve patient outcomes [8,9].

The patient first noticed a stiff neck and dull pain around the shoulders, after which the lumps gradually became increasingly prominent. Although shoulder pain is described as a common manifestation after surgery or radiation of breast cancer, it is rarely reported as the initial symptom of the malignancy itself [10]. The most common presenting symptom is a breast lump, occurring in 83% of patients, whereas musculoskeletal pain is reported in only 0.6% [11]. As for this patient, the right-sided neck stiffness was likely due to compression of the brachial plexus by bulky axillary lymphadenopathies, suggesting thoracic outlet syndrome. Metastases to the right lung apex may also have contributed to referred pain through vagus nerve irritation, while sternal involvement and tumor-associated inflammation could explain the subsequent bilateral shoulder and clavicular pain. Given that this patient reported worsening pain when carrying baggage or raising her arms, the stiff neck was likely largely attributable to brachial plexus compression.

This case highlights an atypical presentation of ER-low breast cancer, where “stiff neck” was the sole symptom at presentation. Remarkably, the patient initially hesitated to mention breast masses, potentially contributing to the diagnostic delay. This phenomenon of denial after noticing a breast lump is well-known [12], with studies showing that 35% of patients with breast symptoms delay seeking medical attention for four weeks or longer [13]. Another survey suggests that women, younger individuals, and those in poor health are more likely to withhold medically relevant information [14]. At the same time, diagnostic delays may also occur on the physician’s side, as breast lumps in young women can be underestimated due to a lower clinical suspicion of malignancy, despite its documented prevalence in this age group [15]. When patients are unwilling to disclose a serious condition, clinicians should adopt an approach that elicits precise cues to the diagnosis by fostering relationships, listening attentively, and lowering the threshold for further cancer investigation.

## Conclusions

Young women with breast malignancy often overlook breast abnormalities or other symptoms, or may avoid reporting them due to embarrassment or anxiety. Therefore, clinicians should avoid relying solely on patient self-report and remain vigilant for subtle or atypical findings through careful physical examination and comprehensive history-taking. Our case report highlights the importance of early detection in breast cancer patients. 
